# Supplementary material for: Phytotoxic Effects and Agricultural Potential of Nanofertilizers: A Study Using Zeolite, Zinc Oxide, and Titanium Dioxide Under Controlled Conditions
Source: J Xenobiot. 2025 Aug 1;15(4):123. doi: 10.3390/jox15040123 (PMC12387226; doi:10.3390/jox15040123)
Supplement: Supplementary file 1 [file jox-15-00123-s001.zip › jox-3705238-supplementary-proof-done.pdf]

Journal of
Xenobiotics

MDPI

Supplementary Material: Phytotoxic Effects and Agricultural Potential of Nanofertilizers: A Case Study Using Zeolite, Zinc Oxide, and Titanium Dioxide Under Controlled Conditions

Ezequiel Zamora-Ledezma, Glenda Leonela Loor Aragundi, Willian Stalyn Guamán Marquines, Michael Anibal Macías Pro, José Vicente García Díaz, Henry Antonio Pacheco Gil, Julián Mauricio Botero Londoño, Mónica Andrea Botero Londoño and Camilo Zamora-Ledezma

Figure S1. Photographic record of experiments. A: Nanomaterials presentation; B: Preparation of petri dishes; C: Toxicant reference application; D-F: Bioassays with nanofertilizers and nanomaterials

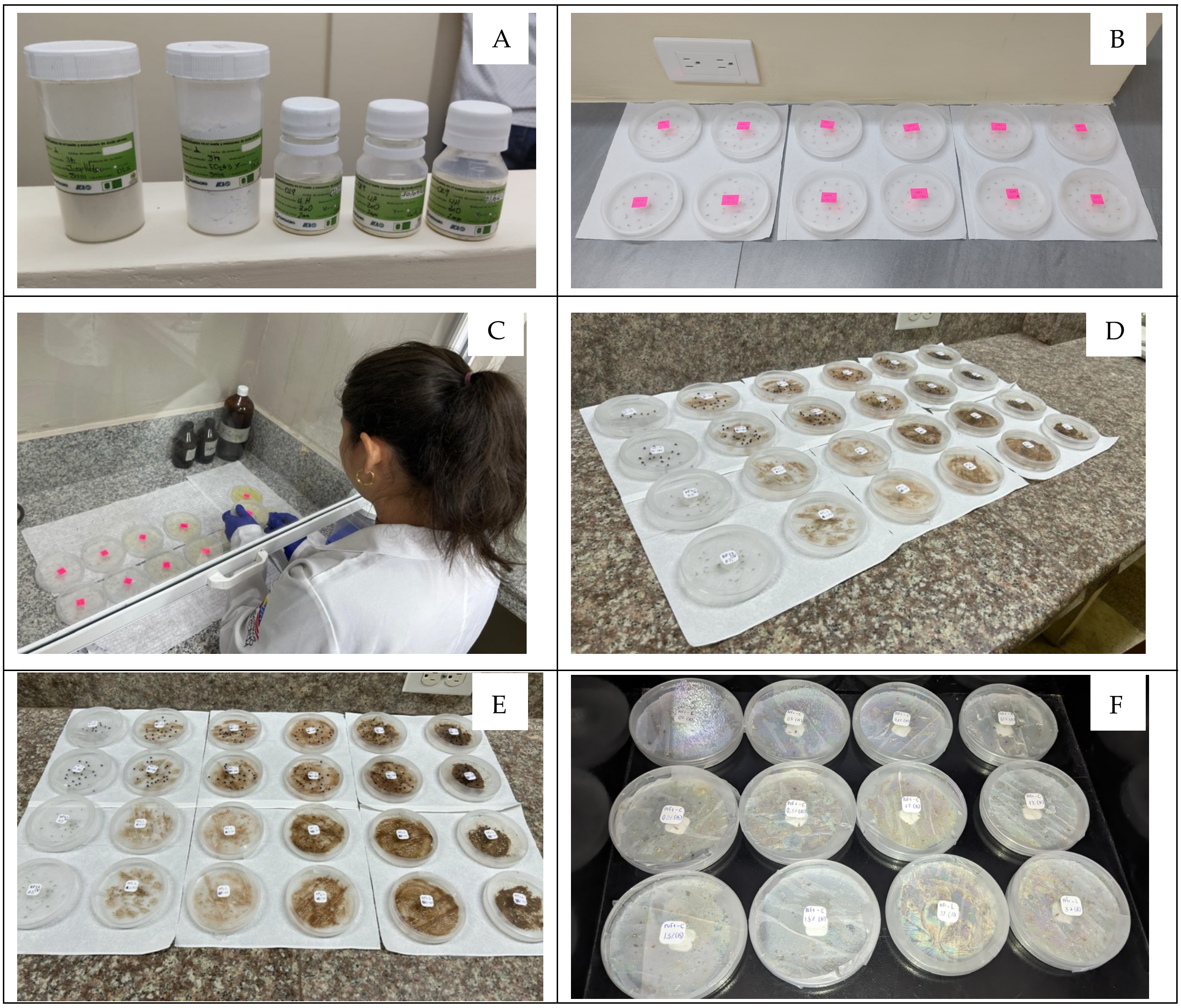

Figure S2. Germination(%) of Lettuce (*Lactuca sativa*) seeds exposed to different concentrations of potassium dichromate.

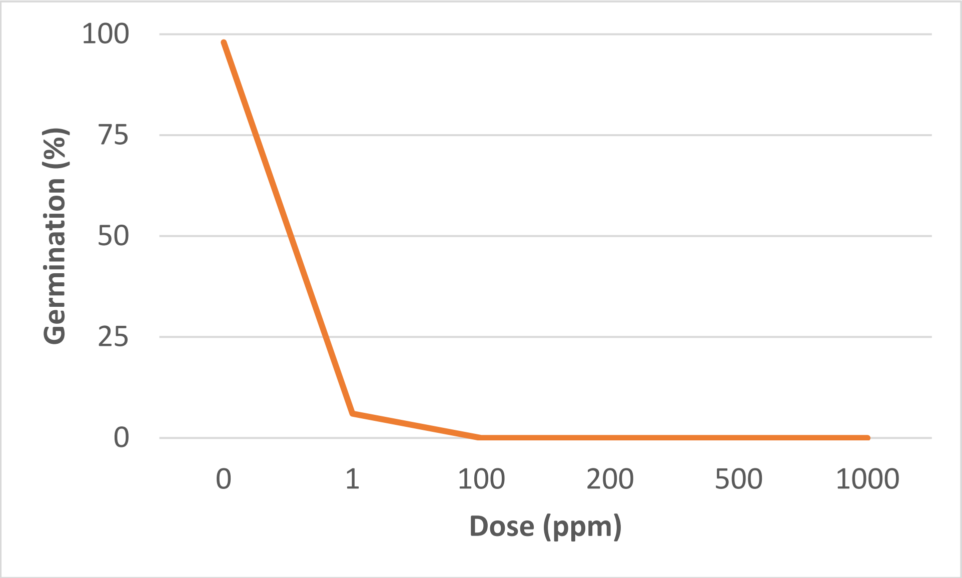

Figure S3. EC_{50} of Lettuce (*Lactuca sativa*) seeds exposed to different concentrations of potassium dichromate.

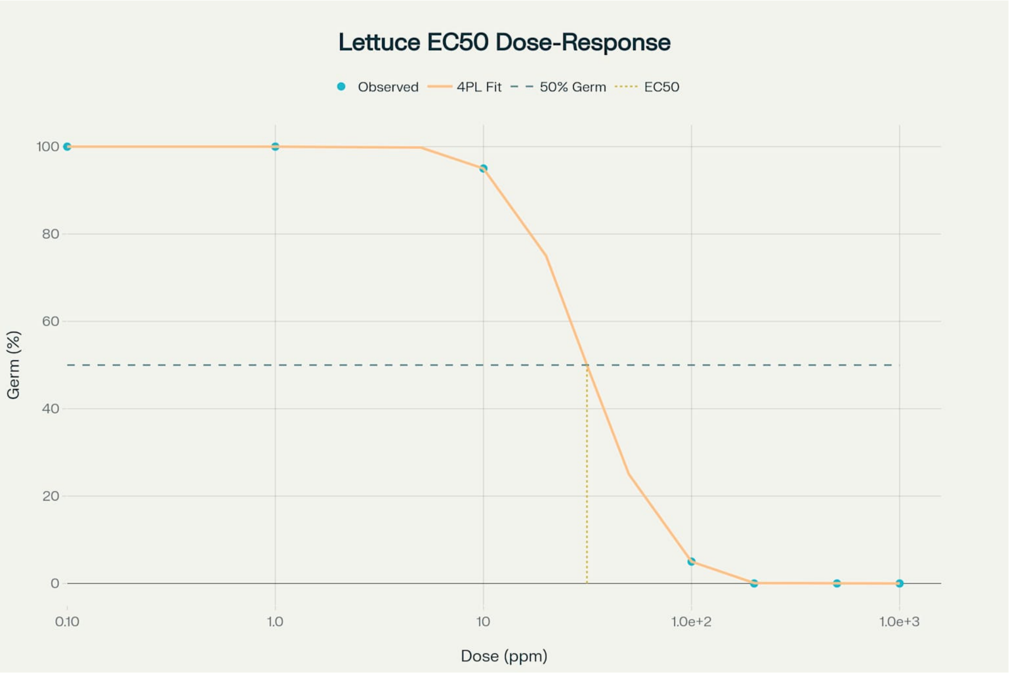

Table S1. Summary of criterion and results for EC_{50} of Lettuce (*Lactuca sativa*) seeds exposed to different concentrations of potassium dichromate.

Parameter	Value	Interpretation
**EC50**	31.4 ppm	Concentration causing 50% germination inhibition
**95% Confidence Interval**	27.8 - 35.1 ppm	Statistical precision range
**Statistical Model**	4-parameter logistic	Most robust for sigmoidal dose-response
**Control Germination**	100%	Excellent (≥90% required)
**Response Pattern**	Sigmoidal	Clear dose-dependent relationship
**Goodness of Fit (R^2)**	1.0000	Excellent model fit
**Bioassay Quality**	Valid	Meets international standards

Table S2. Number of germinated seeds in the different doses and treatments. NF1: nanofertilizer 1, NF2: nanofertilizer 2, NF3: nanofertilizer 3.

Dose (%)	NF1 (#)	NF2 (#)	NF3 (#)
0	50	49	50
0.5	45	46	41
0.9	40	39	38
1	39	38	24
2	31	16	2
3	0	0	0

Table S3. Number of germinated seeds in the different doses and treatments of nanomaterials. NM1: nanomaterial 1, NM2: nanomaterial 2, NM3: nanomaterial 3.

Dose (%)	NM1 (#)	NM2 (#)	NM3 (#)
0	40	44	44
10	36	42	40
20	31	37	-
30	28	28	40
40	28	21	32
50	0	6	22
60	-	-	0

Table S4. Germination rates, Root length and Hypocotyl length in the different doses and treatments of nanofertilizers. NF1: nanofertilizer 1, NF2: nanofertilizer 2, NF3: nanofertilizer 3.

**Treatments:**	**NF1**		**NF2**		**NF3**	
Dose	***Germination (%)***					
	Mortality	*Surv.*	*Mortality*	*Surv.*	*Mortality*	*Surv.*
0	0	100	2	98	0	100
0.5	10	90	8	92	18	82
0.9	20	80	22	78	22	78
1	22	78	24	76	52	48
2	38	62	68	32	96	4
3	100	0	100	0	100	0
Dose	***Root length (mm)***					
	Mean	*SD*	*Mean*	*SD*	*Mean*	*SD*
0	7.01	2.65	8.74	3.65	8.51	3.21
0.5	3.91	1.18	5.63	2.32	4.32	1.61
0.9	2.50	0.86	3.07	0.99	3.56	0.85
1	2.86	0.87	3.01	1.40	4.37	1.27
2	1.96	0.64	1.83	0.42	1.75	1.06
3	0.00	0.00	0.00	0.00	0.00	0.00
Dose	***Hypocotyl length (mm)***					
	Mean	*SD*	*Mean*	*SD*	*Mean*	*SD*
0	9.38	3.53	9.40	3.10	8.83	3.79
0.5	9.74	4.16	13.48	5.75	13.17	5.77
0.9	7.06	2.64	7.29	3.00	10.83	3.48
1	7.21	2.00	6.09	2.50	12.73	4.45
2	2.42	1.58	3.71	1.48	0.00	0.00
3	0.00	0.00	0.00	0.00	0.00	0.00

Table S5. Germination rates, Root length and Hypocotyl length in the different doses and treatments of nanomaterials. NM1: nanomaterial 1, NM2: nanomaterial 2, NM3: nanomaterial 3.

**Treatments:**	**NM1**		**NM2**		**NM3***	
Dose	***Germination (%)***					
	Mortality	*Surv.*	*Mortality*	*Surv.*	*Mortality*	*Surv.*
0	20	80	12	88	12	88
10	28	72	16	84	20	80
20	38	62	26	74	20	80
30	44	56	44	56	36	64
40	44	56	58	42	56	44
50	100	0	88	12	100	0
Dose	***Root length (mm)***					
	Mean	*SD*	*Mean*	*SD*	*Mean*	*SD*
0	3.96	1.61	4.37	1.76	4.37	1.76
10	2.83	0.78	3.47	2.03	4.45	2.51
20	2.36	0.96	3.74	1.59	4.57	1.77
30	2.23	0.54	3.21	1.30	4.22	1.78
40	3.03	1.15	2.74	0.74	5.85	1.88
50	0.00	0.00	1.86	1.24	0.00	0.00
Dose	***Hypocotyl length (mm)***					
	Mean	*SD*	*Mean*	*SD*	*Mean*	*SD*
0	2.98	1.97	4.35	2.44	4.35	2.44
10	2.72	1.29	3.25	2.06	5.32	2.99
20	2.01	1.10	3.25	1.78	4.70	2.18
30	2.37	0.98	3.99	2.30	5.01	2.35
40	3.13	2.33	2.65	1.19	5.59	2.63
50	0.00	0.00	1.86	1.24	0.00	0.00

**: doses for NM3 were always 0%, 10%, 20%, 40%, 50% and 60%*

Table S6. Detailed dose-response data for germination rate (%) of *Lactuca sativa* under nanofertilizer exposure, based on one-way ANOVA. (corresponding to Fig. 1A).

Source of variation	GL	SC (Ajusted)	MC (Ajusted)	F value	P value
Treatment	2	1650.7	825.33	17.36	0.000
Error	18	856.0	47.56		
Total	35	47916.0			

NF1 treatment

Grouping based on fisher's LDS test (95% confidence level)

Dose	N	Neam	Grouping
0.0	2	100	A
0.5	2	90	AB
0.9	2	80	BC
1.0	2	78	C
2.0	2	62	D
3.0	2	0	E

NF2 treatment

Grouping based on fisher's LDS test (95% confidence level)

Dose	N	Neam	Grouping
0.0	2	98	A
0.5	2	92	A
0.9	2	78	A
1.0	2	76	A
2.0	2	32	B
3.0	2	0	C

NF3 treatment

Grouping based on fisher's LDS test (95% confidence level)

Dose	N	Neam	Grouping
0.0	2	100	A
0.5	2	82	B
0.9	2	78	B
1.0	2	48	C
2.0	2	4	D
3.0	2	0	D

Table S7. Detailed dose-response data for germination rate (%) of *Lactuca sativa* under nanomaterials exposure, based on one-way ANOVA. (corresponding to Fig. 1B-1C).

Source of variation	GL	SC (Ajusted)	MC (Ajusted)	F value	P value
Treatment	2	1494	747.13	6.06	0.007
Error	27	3330	123.32		
Lack of adjustment	9	1642	182.41	1.95	0.110
Pure error	18	1688	93.78		
Total	35	29164			

NM1 treatment

Grouping based on fisher's LDS test (95% confidence level)

Dose	N	Neam	Grouping
0	2	80	A
10	2	72	AB
20	2	62	BC
30	2	56	C
40	2	56	C
50	2	0	D

NM2 treatment

Grouping based on fisher's LDS test (95% confidence level)

Dose	N	Neam	Grouping
0	2	88	A
10	2	84	A
20	2	74	AB
30	2	56	BC
40	2	42	C
50	2	12	D

NM3 treatment

Grouping based on fisher's LDS test (95% confidence level)

Dose	N	Neam	Grouping
0	2	88	A
10	2	80	A
20	2	80	A
40	2	64	AB

50	2	44	B
60	2	0	C

Table S8. Detailed dose-response data for root elongation of *Lactuca sativa* under exposure to nanofertilizers, based on a one-way ANOVA (corresponding to Figure 1D).

Source of variation	GL	SC (Ajusted)	MC (Ajusted)	F value	P value
Tratamiento	2	2.765	1.3825	2.72	0.093
Error	18	9.143	0.5079		
Total	35	242.070			

Table S9. Detailed dose-response data for root elongation of *Lactuca sativa* under exposure to nanomaterials, based on a one-way ANOVA (corresponding to Figure 1E-1F).

Source of variation	GL	SC (Ajusted)	MC (Ajusted)	F value	P value
Treatment	2	46.170	23.0849	25.85	0.000
Error	27	24.116	0.8932		
Lack of adjustment	9	19.210	2.1344	7.83	0.000
Pure error	18	4.906	0.2726		
Total	35	110.262			

NM1 treatment

Grouping based on fisher's LDS test (95% confidence level)

Doses	N	Mean	Grouping
0	2	3.56316	A
10	2	2.34833	A
20	2	2.34833	A
30	2	2.45313	A
40	2	3.02500	A
50	2	0.00000	B

NM2 treatment

Grouping based on fisher's LDS test (95% confidence level)

Doses	N	Mean	Grouping
0	2	4.20476	A
10	2	2.81331	B
20	2	2.34833	B
30	2	2.19479	B
40	2	3.02500	B
50	2	-0.00000	C

NM3 treatment

Grouping based on fisher's LDS test (95% confidence level)

Doses	N	Mean	Grouping
0	2	4.38002	A
10	2	5.51233	A
20	2	4.69192	A
40	2	5.00938	A
50	2	5.63162	A
60	2	0.00000	B

Table S10. Detailed dose-response data for hypocotyl elongation of *Lactuca sativa* under exposure to nanofertilizers, based on a one-way ANOVA (corresponding to Figure 1G).

**Source of variation**	**GL**	**SC (Ajusted)**	**MC (Ajusted)**	**F value**	**P value**
Treatment	2	16.41	8.205	3.25	0.062
Error	18	45.41	2.523		
Total	35	795.32			

Table S11. Detailed dose-response data for hypocotyl elongation of *Lactuca sativa* under exposure to nanomaterials, based on a one-way ANOVA (corresponding to Figure 1H-1I).

**Source of variation**	GL	SC (Ajusted)	MC (Ajusted)	F value	P value
Treatment	2	53.739	26.8697	26.76	0.000
Error	27	27.108	1.0040		
Lack of adjustment	9	21.139	2.3488	7.08	0.000
Pure error	18	5.968	0.3316		
Total	35	119.442			

NM1 treatment

Grouping based on fisher's LDS test (95% confidence level)

Doses	N	Mean	Grouping

0	2	2.97093	A
10	2	2.71846	AB
20	2	2.01333	B
30	2	2.33333	AB
40	2	3.12500	A
50	2	0.00000	D

NM2 treatment

Grouping based on fisher's LDS test (95% confidence level)

Doses	N	Mean	Grouping
0	2	4.34348	A
10	2	3.25568	BC
20	2	3.25643	BC
30	2	4.01615	AB
40	2	2.63455	C
50	2	0.00000	D

NM3 treatment

Grouping based on fisher's LDS test (95% confidence level)

Doses	N	Mean	Grouping
0	2	4.34348	A
10	2	5.49400	A
20	2	4.69192	A
40	2	5.77188	A
50	2	5.63162	A
60	2	0.00000	B

Table S12. Relative germination percentage (RGP) and Relative radicle growth (RRG) in the different doses and treatments of nanofertilizers. NM1: nanomaterial 1, NM2: nanomaterial 2, NM3: nanomaterial 3, RGPD: Relative germination percentage in doses, RGPC: Relative germination percentage in controls, RGD: Relative radicle growth in doses, RGC: Relative radicle growth in controls.

Dose %	Relative germination percentage (RGP)			Relative radicle growth (RRG)		
	RGPD (%)	*RGPC (%)*	*RGP (%)*	*RGD (mm)*	*RGC (mm)*	*RRG (%)*
			NF1			
0	50	50	100	7.01	7.01	100
0.5	45	50	90	3.91	7.01	56
0.9	40	50	80	2.50	7.01	36
1	39	50	78	2.86	7.01	41
2	31	50	62	1.96	7.01	28
3	0	50	0	0.00	7.01	0
			NF2			
0	49	49	100	8.74	8.74	100
0.5	46	49	94	5.63	8.74	64
0.9	39	49	80	3.07	8.74	35
1	38	49	78	3.01	8.74	34
2	16	49	33	1.83	8.74	21
3	0	49	0	0.00	8.74	0
			NF3			
0	50	50	100	8.51	8.51	100
0.5	41	50	82	4.32	8.51	51
0.9	39	50	78	3.56	8.51	42
1	24	50	48	4.37	8.51	51
2	2	50	4	1.75	8.51	21
3	0	50	0	0.00	8.51	0

Table S13. Relative germination percentage (RGP) and Relative radicle growth (RRG) in the different doses and treatments of nanofertilizers. NM1: nanomaterials 1, NM2: nanofertilizer 2, NM3: nanofertilizer 3, RGPD: Relative germination percentage in doses, RGPC: Relative germination percentage in controls, RGD: Relative radicle growth in doses, RGC: Relative radicle growth in controls.

Dose %	Relative germination percentage (RGP)			Relative radicle growth (RRG)		
	RGPD (%)	*RGPC (%)*	*RGP (%)*	*RGD (mm)*	*RGC (mm)*	*RRG (%)*
			NM1			
0	40	40	100	1.61	1.61	100
10	36	40	90	0.78	1.61	49
20	31	40	78	0.96	1.61	60
30	28	40	70	0.54	1.61	34
40	28	40	70	1.15	1.61	72
50	0	40	0	0.00	1.61	0
			NM2			
0	44	44	100	1.76	1.76	100
10	42	44	95	2.03	1.76	115
20	37	44	84	1.59	1.76	90
30	28	44	64	1.30	1.76	74
40	21	44	48	0.74	1.76	42
50	6	44	14	1.24	1.76	71
			NM3			
0	44	44	100	4.37	4.37	100
10	40	44	91	4.45	4.37	102
20	40	44	91	4.57	4.37	105
40	32	44	73	4.22	4.37	97
50	22	44	50	5.85	4.37	134
60	0	44	0	0.00	4.37	0
